# Computer-aided drug design

**DOI:** 10.1186/s13065-023-00939-w

**Published:** 2023-03-24

**Authors:** Abdulilah Ece

**Affiliations:** Department of Pharmaceutical Chemistry, Faculty of Pharmacy, Biruni University, Istanbul, Türkiye

## Abstract

Computer-Aided Drug Design tools are now an indispensable part of drug discovery that have made key contributions to the development of drugs. In this editorial, I briefly provide an overview of CADD emphasizing its potential and invite authors from academia and the pharmaceutical and biotechnology sector to present their research in this collection.

## Introduction

The biological origin of a disease underlies the modern treatment of a particular health issue. In early times, natural products were used as a cure or to alleviate symptoms. Advancements in science and technology led to the discovery of macromolecules that played a direct or indirect role in a specific disease. In turn, these discoveries guided chemists to design and synthesize novel bioactive compounds increasingly more active than their predecessors. However, getting a druggable compound to market consists of several steps, many challenges and a tremendous amount of work, time and budget. Between 2009 and 2018 the average cost of drug design and discovery was reported to be up to $2.8 billion [1]. Hence, new approaches are necessary to overcome these economic and time-related challenges.

Computer-Aided Drug Design (CADD) strategies have become indispensable tools in modern drug discovery and development. Besides academia, large and small-sized pharmaceutical and biotechnology companies have been using intelligent software to assist in the discovery or optimization of bioactive compounds. Several drugs were discovered and/or optimized using CADD technologies in different stages. This includes, but is not limited to, Aliskiren, Boceprevir, Captopril, Dorzolamide, Nolatrexed, Oseltamivir, Rupintrivir, Saquinavir and Zanamivir [2].

CADD can be roughly divided into two categories: structure-based drug design (SBDD) and ligand-based drug design (LBDD). If a 3D structure of the target macromolecule is not known or cannot be constructed using homology modelling, then we are left with LBDD tools. As an example, the quantitative structure–activity relationship (QSAR) has been used for decades. In this method, an equation is constructed which relates biological activity as a function of predicted parameters/descriptors of ligands [3]. Consequently, it allows us to predict the activity of a compound prior to wet lab experiments. A good QSAR equation depends on the reliability of data (correct homolog/analog series and activity ranges) and the statistics used to test and build the equation. Another frequently used LBDD method is pharmacophore studies. A pharmacophore defines molecular features (e.g., hydrogen bond acceptor/donor, aromatic, hydrophobic, ionizable etc.) that are critical in molecular recognition of ligands by a macromolecule. A good pharmacophore can also be generated using SBDD. The features required in binding of ligands with critical amino acid residues are constructed from the binding site of the target biological macromolecule. A pharmacophore can be used as a 3D query to find drug candidates for further optimization and testing, from a database consisting of millions (or even billions) of molecules (virtual screening) while only thousands of compounds are generally screened using molecular docking. Molecular dynamics simulations, as a part of SBDD, are often used to conduct a simulation of biomolecules to obtain information about the conformations, fluctuations and dynamics of a macromolecular system [4]. Although the history of it goes back to 1950 [5], researchers have only been recently employing artificial intelligence (AI) on CADD [6]. In parallel with the advancement of high-performance computing, many complex problems might easily be solved with the help of AI using a combination of CADD tools.

Different algorithms are used to calculate the structural properties and conformations of the small molecules or macromolecules in question [7]. Equations used in molecular mechanics are based on the laws of classical physics. Molecular mechanics simplifies compounds as atoms being spheres and bonds being springs. With this approach, the many interactions and energies of large systems (e.g., proteins) can easily be calculated with less computational time and resources because electrons are not taken into account. Conversely, interactions of electrons with the nuclei of the compounds should be considered in small systems which require the application of quantum mechanics calculations. Quantum mechanics are based on quantum physics and very accurate electronic properties can be predicted using the appropriate level of theory and basis set. For instance, electron potential maps and frontier molecular orbitals and several parameters related with to them (chemical hardness, softness, ionization potential etc.) can be calculated. In many cases, a combination of several techniques has to be used and methods are frequently adjusted for different drug design projects [8–10].

CADD remains a field of rapid development. Unfortunately, CADD suffers from cognitive dissonance (bias to seek consonance) and also sloppy research that results from the lack of proper training. Each step should be validated before predicting any property or a complex system. Internal validation (RMSD in molecular docking), cross-validation, fisher validation and student t-test (in 2D or 3D QSAR), robust initial enhancement, receiver operating characteristic, Boltzman enhanced discrimination of the receiver operating characteristic curve and enrichment factor etc. (in virtual screening) are just a few validations/statistical parameters that have to be evaluated first [8].

Thus, in order to draw attention to this hot topic and to provide researchers to present their research, I am glad to welcome submissions to a BMC Chemistry collection devoted to CADD. More details can be found at: https://www.biomedcentral.com/collections/cadd. We invite authors spanning the fields of chemistry, biology, medicine, computational design and beyond from both academics and pharma companies to present their research as research articles or reviews. We are hoping that this collection will bring together recent advances in CADD and highlight the vital role of computational sciences on drug discovery and design processes to shorten the cycle of drug discovery.

## Data Availability

Not applicable.

## Abbreviations

AI: Artificial intelligence
CADD: Computer-Aided Drug Design
LBDD: Ligand-based drug design
SBDD: Structure-based drug design
QSAR: Quantitative structure activity relationship

## References

[1] Wouters OJ, McKee M, Luyten J (2020). Estimated research and development investment needed to bring a new medicine to market, 2009–2018. JAMA.

[2] Talele TT, Khedkar SA, Rigby AC (2010). Successful applications of computer aided drug discovery: moving drugs from concept to the clinic. Curr Top Med Chem.

[3] Ece A, Sevin F (2010). Exploring QSAR on 4-cyclohexylmethoxypyrimidines as antitumor agents for their inhibitory activity of cdk2. Lett Drug Des Discovery.

[4] Godwin RC, Melvin R, Salsbury FR (2016). Molecular dynamics simulations and computer-aided drug discovery. Computer-aided drug discovery.

[5] Turing A (1950). Computing machinery and intelligence. Mind.

[6] Yang X, Wang Y, Byrne R, Schneider G, Yang S (2019). Concepts of artificial intelligence for computer-assisted drug discovery. Chem Rev.

[7] Patrick GL (2013). An introduction to medicinal chemistry.

[8] Ece A (2020). Towards more effective acetylcholinesterase inhibitors: a comprehensive modelling study based on human acetylcholinesterase protein–drug complex. J Biomol Struct Dyn.

[9] Er M, Abounakhla AM, Tahtaci H, Bawah AH, Çınaroğlu SS, Onaran A (2018). An integrated approach towards the development of novel antifungal agents containing thiadiazole: synthesis and a combined similarity search, homology modelling, molecular dynamics and molecular docking study. Chem Cent J.

[10] Ece A, Pejin B (2015). A computational insight into acetylcholinesterase inhibitory activity of a new lichen depsidone. J Enzyme Inhib Med Chem.

